# Supplementary material for: When dialects collide: how socioeconomic mixing affects language use
Source: EPJ Data Sci. 2025 Jul 10;14(1):47. doi: 10.1140/epjds/s13688-025-00563-9 (PMC12245997; doi:10.1140/epjds/s13688-025-00563-9)
Supplement: Supplementary file 1 — (PDF 1.6 MB) [file 13688_2025_563_MOESM1_ESM.pdf]

Supplementary Material:
When Dialects Collide:
How Socioeconomic Mixing Affects Language Use

Thomas Louf,[1,2*] José J. Ramasco,[1] David Sánchez,[1] Márton Karsai[3,4]

[1]Institute for Cross-Disciplinary Physics and Complex Systems IFISC (UIB-CSIC)
Palma de Mallorca, Spain
[2]Fondazione Bruno Kessler
Povo (TN), Italy
[3]Department of Network and Data Science, Central European University
Vienna, Austria
[4]National Laboratory for Health Security, HUN-REN Alfréd Rényi Institute of Mathematics
Budapest, Hungary
*To whom correspondence should be addressed; E-mail: tlouf@fbk.eu.

Contents

S1 Description of the dataset 4

S2 Statistics for the categories of standard language rules 4

S3 Metropolitan areas' definition 4

S4 Assortativity's dependence on the number of classes 5

S5 Influence of multilingualism 5

S6 Analytic results for our model of variety adoption 5

S6.1 Notation 5

S6.2 Assumptions 6

S6.3 Deriving the master equations 7

S6.4 Case of equal populations and mobility 10

S6.5 Coexistence solution 10

List of Figures

S1 Twitter population map in England and Wales. 12

S2 The influence of the number of classes on the computed assortativity. 13

List of Tables

S1 Summary statistics about each category of rules defined by LanguageTool. . . . 14

S2 Ten most frequently detected grammar rules. 14

S3 Definition of the metropolitan areas used in this study 15

S4 Summary statistics of our Twitter corpus. 16
S5 Proportions of multilinguals among our identified residents and their correlation with grammar deviations. 16

S1 Description of the dataset

Table S4 gives some summary statistics of our filtered Twitter dataset, obtained after going through all the pre-processing steps described in the main text. These show some variation from one metropolitan area to another, but, reassuringly, the user averages are quite consistent across the board.

We also show a map presenting the population of each MSOA of England and Wales in Fig. S1.

S2 Statistics for the categories of standard language rules

Table S1 gives the number of matches for the rules of each category defined by LanguageTool on our filtered corpus, as well as the computed Pearson r correlation of their user-averaged frequencies with the average net income in the MSOAs of England and Wales. The very high number of typographical deviations is mostly due to the presence of extra whitespaces induced by our filtering of URLs, hashtags and mentions.

We subsequently focused on grammar features, which seem the most anti-correlated with net income. The top ten rules from that category are given in Table S2, to give an idea of the kind of rules that served us as a proxy to quantify deviations from the standard variety.

More information about LanguageTool's rules and categories can be found at `https://community.languagetool.org/rule/list`.

S3 Metropolitan areas' definition

Table S3 details explicitly the areas included in our definitions of the eight metropolitan areas studied throughout this work. Clearly, all areas show a similar number of tweets, tokens and frequencies of non-standard features per user, which makes it possible to make comparisons

between cities reliably.

S4 Assortativity's dependence on the number of classes

In the main text, we give assortativity values in our eight metropolitan areas that were computed after defining five socioeconomic classes. We show in Fig. S2 the values of the assortativity for three and ten classes, which show the robustness of our measurement when the number of classes is not too small.

S5 Influence of multilingualism

In order to uncover a potential impact of the potential multilingual backgrounds of our home-located users on our study, we performed the following analysis. We gathered all the tweets from our identified residents and performed a language detection on each of them. To do so, we first cleared them of hashtags, URLs and mentions, and then kept those tweets which retained at least 4 words. All tweets which passed this threshold are then passed into Chromium's Compact Language Detector (CLD), which, for each tweet, gives us a language and the confidence of the algorithm. We therefore only kept those with a confidence above 90%, to then count for each user the number of times they tweeted in different languages. For each user, we only keep languages that either appear in at least 3 tweets or 10% of them, as some may occasionally use a translator or quote someone else in a language they cannot speak. The results are presented in Table S5.

S6 Analytic results for our model of variety adoption

S6.1 Notation

Let us introduce the following notation:

- Σ the set of SE classes in a population:

$$\Sigma = \{\sigma_k \mid k \in [1, n_\sigma]\}\,, \tag{S1}$$

- C the set of cells of residence:

$$C = \{c_i \mid i \in [1, n_C]\}\,, \tag{S2}$$

- $N_{c,\sigma}$ the number of residents of cell c with class σ,
- $N_c \equiv \sum_\sigma N_{c,\sigma}$ the population of cell c,
- $N_\sigma \equiv \sum_c N_{c,\sigma}$ the population of class σ,
- $N \equiv \sum_{c,\sigma} N_{c,\sigma}$ the total population,
- $M_{i,j}$ the probability for a resident of c_i to move to c_j.

S6.2 Assumptions

Let there be only two cells: $n_C = 2$, and two SE classes: $n_\sigma = 2$, completely separated, with the whole σ_1 population in c_1 and the whole σ_2 population in c_2:

$$\begin{aligned} N_1 &\equiv N_{c_1,\sigma_1} = N_{\sigma_1}, \\ N_2 &\equiv N_{c_2,\sigma_2} = N_{\sigma_2}. \end{aligned} \tag{S3}$$

This implies that the $M_{i,j}$ can be summarized with just two values, each corresponding to a class:

$$\begin{aligned} M_1 &\equiv M_{1,2} = 1 - M_{1,1}, \\ M_2 &\equiv M_{2,1} = 1 - M_{2,2}. \end{aligned} \tag{S4}$$

Let us consider two varieties 1 and 2. This could be the use of standard language (1 means they do, 2 means they do not). Now let us introduce an intrinsic prestige s for the variety 2, such that:

$$\begin{aligned} P(1 \to 2) &\propto s, \\ P(2 \to 1) &\propto 1 - s. \end{aligned} \tag{S5}$$

Without loss of generality, let us assume $s > 1/2$, meaning 2 is more prestigious than 1. And let us introduce an asymmetric attachment of each group for their own variety, q_1 and q_2:

$$\begin{aligned} &P(2 \to 1 \mid \sigma = \sigma_1) \propto q_1 > 1/2, \qquad &&P(1 \to 2 \mid \sigma = \sigma_1) \propto 1 - q_1, \\ &P(1 \to 2 \mid \sigma = \sigma_2) \propto q_2, &&P(2 \to 1 \mid \sigma = \sigma_2) \propto 1 - q_2. \end{aligned} \tag{S6}$$

So 2 is more prestigious than 1, but individuals of class σ_1 prefer 1.

We will write p_1 the proportion of individuals of class 1 speaking non-standard (variety 1), and p_2 the proportion of individuals of class 2 speaking standard (variety 2). Then, working in mean-field, one can write the following master equations:

$$\begin{aligned} \frac{\mathrm{d}p_1}{\mathrm{d}t} &= (1 - p_1)P(2 \to 1 \mid \sigma = \sigma_1) - p_1 P(1 \to 2 \mid \sigma = \sigma_1) \\ \frac{\mathrm{d}p_2}{\mathrm{d}t} &= (1 - p_2)P(1 \to 2 \mid \sigma = \sigma_2) - p_2 P(2 \to 1 \mid \sigma = \sigma_2) \end{aligned} \tag{S7}$$

S6.3 Deriving the master equations

Following the definitions in Eqs. (S5) and (S6) of the influence of s, q_1 and q_2, the transition probabilities can be written as follows:

$$\begin{aligned} P(1 \to 2 \mid \sigma = \sigma_1, c_t = c_j) &= s(1 - q_1)P(v_{t-1} = 2 \mid c_t = c_j) \\ P(1 \to 2 \mid \sigma = \sigma_2, c_t = c_j) &= s q_2 P(v_{t-1} = 2 \mid c_t = c_j) \\ P(2 \to 1 \mid \sigma = \sigma_1, c_t = c_j) &= (1 - s) q_1 P(v_{t-1} = 1 \mid c_t = c_j) \\ P(2 \to 1 \mid \sigma = \sigma_2, c_t = c_j) &= (1 - s)(1 - q_2)P(v_{t-1} = 1 \mid c_t = c_j) \end{aligned} \tag{S8}$$

with $P(1 \to 2) \equiv P(v_t = 2 \mid v_{t-1} = 1)$, $P(2 \to 1) \equiv P(v_t = 1 \mid v_{t-1} = 2)$, t denoting the current time step, $P(v_{t-1} = v \mid c_t = c_j)$ the probability to pick an individual who used variety

v at $t-1$ and who is at cell c_j at t. Decomposing it by SE class, and using Bayes' rule, we get:

$$\begin{aligned} &P(v_{t-1} = v \mid c_t = c_j) \\ &= \sum_k P(v_{t-1} = v, \sigma = \sigma_k \mid c_t = c_j) \\ &= \sum_k P(v_{t-1} = v, \sigma = \sigma_k) \cdot \frac{P(c_t = c_j \mid v_{t-1} = v, \sigma = \sigma_k)}{P(c_t = c_j)} \\ &= \sum_k P(v_{t-1} = v \mid \sigma = \sigma_k) \cdot P(\sigma = \sigma_k) \cdot \frac{P(c_t = c_j \mid v_{t-1} = v, \sigma = \sigma_k)}{P(c_t = c_j)}. \end{aligned} \tag{S9}$$

Let us introduce $p_{v,\sigma_k} \equiv P(v_{t-1} = v \mid \sigma = \sigma_k)$ to simplify equations further, which summarise the state of the system at the previous step. Also, since they satisfy $\sum_v p_{v,\sigma_k} = 1$, we will only write in terms of $p_1 \equiv p_{1,1}$ and $p_2 \equiv p_{2,2}$. We also have $P(\sigma = \sigma_k) = \frac{N_{\sigma_k}}{\sum_k N_{\sigma_k}}$. The final term in the product above is related to the mobility of the different SES classes. Indeed, aligning the indices of the SE class with the one of their cell of residence, and using the fact that the random variable C_t, which represents the cell where an individual will be encountered at step t, is independent from V_{t-1}, which represents an individual's variety usage at the previous step, we have:

$$P(c_t = c_j \mid v_{t-1} = v, \sigma = \sigma_k) = P(c = c_j \mid \sigma = \sigma_k) = M_{k,j}, \tag{S10}$$

and

$$P(c_t = c_j) = \sum_k P(c = c_j \mid \sigma = \sigma_k) P(\sigma = \sigma_k) = \sum_k M_{k,j} \frac{N_{\sigma_k}}{\sum_k N_{\sigma_k}}. \tag{S11}$$

Let us now introduce

$$m_{k,j} \equiv \frac{N_{\sigma_k} M_{k,j}}{\sum_{k'} N_{\sigma_{k'}} M_{k',j}}, \tag{S12}$$

which satisfy $\sum_k m_{k,j} = 1$ (unlike the $M_{k,j}$ that satisfy $\sum_j M_{k,j} = 1$). It is simply the expected proportion of individuals that will appear in j that have status k. We can then write

$$P(v_{t-1} = v \mid c_t = c_j) = \sum_k p_{v,\sigma_k} m_{k,j}. \tag{S13}$$

We will further abuse notation and only use $m_1 \equiv m_{1,2}$ and $m_2 \equiv m_{2,1}$, which summarize how mobile each group is. Let us now rewrite (S13) in terms of m_1, m_2, p_1 and p_2 only:

$$\begin{aligned}
P(v_{t-1} = 2 \mid c = c_1) &= (1 - m_2)(1 - p_1) + m_2 p_2 \\
P(v_{t-1} = 2 \mid c = c_2) &= m_1(1 - p_1) + (1 - m_1)p_2 \\
P(v_{t-1} = 1 \mid c = c_1) &= (1 - m_2)p_1 + m_2(1 - p_2) \\
P(v_{t-1} = 1 \mid c = c_2) &= m_1 p_1 + (1 - m_1)(1 - p_2)
\end{aligned} \tag{S14}$$

We can subsequently write the forms in (S8) in terms of these four variables only. But what we actually want is to write $P(v_t = v \mid v_{t-1} = v', \sigma = \sigma_k)$ for $v' \neq v$. Decomposing this one by cell, we can get the following:

$$\begin{aligned}
&P(v_t = v \mid v_{t-1} = v', \sigma = \sigma_k) \\
&\quad = \sum_j P(c = c_j \mid \sigma = \sigma_k) \cdot P(v_t = v \mid v_{t-1} = v', \sigma = \sigma_k, c_t = c_j) \\
&\quad = \sum_j M_{k,j} P(v_t = v \mid v_{t-1} = v', \sigma = \sigma_k, c_t = c_j).
\end{aligned} \tag{S15}$$

Finally, inserting (S8) into (S15), we get:

$$\begin{aligned}
P(1 \to 2 \mid \sigma = \sigma_1) &= s(1 - q_1)[(1 - M_1)P(v_{t-1} = 2 \mid c = c_1) \\
&\qquad + M_1 P(v_{t-1} = 2 \mid c = c_2)] \\
P(1 \to 2 \mid \sigma = \sigma_2) &= s q_2 [M_2 P(v_{t-1} = 2 \mid c = c_1) \\
&\qquad + (1 - M_2)P(v_{t-1} = 2 \mid c = c_2)] \\
P(2 \to 1 \mid \sigma = \sigma_1) &= (1 - s)q_1[(1 - M_1)P(v_{t-1} = 1 \mid c = c_1) \\
&\qquad + M_1 P(v_{t-1} = 1 \mid c = c_2)] \\
P(2 \to 1 \mid \sigma = \sigma_2) &= (1 - s)(1 - q_2)[M_2 P(v_{t-1} = 1 \mid c = c_1) \\
&\qquad + (1 - M_2)P(v_{t-1} = 1 \mid c = c_2)]
\end{aligned} \tag{S16}$$

S6.4 Case of equal populations and mobility

In the following, we will assume $N_{\sigma_1} = N_{\sigma_2}$, which implies that $m_{k,j} = \frac{M_{k,j}}{\sum_{k'} M_{k',j}}$. If we assume equal mobility, introducing $M \equiv M_1 = M_2$, we have $m_1 = m_2 = M$, and it follows that:

$$\begin{aligned}
P(1 \to 2 \mid \sigma = \sigma_1) &= s(1 - q_1)\left[M^*(p_1 + p_2 - 1) + 1 - p_1\right] \\
P(1 \to 2 \mid \sigma = \sigma_2) &= sq_2\left[M^*(1 - p_1 - p_2) + p_2\right] \\
P(2 \to 1 \mid \sigma = \sigma_1) &= (1 - s)q_1\left[M^*(1 - p_1 - p_2) + p_1\right] \\
P(2 \to 1 \mid \sigma = \sigma_2) &= (1 - s)(1 - q_2)\left[M^*(p_1 + p_2 - 1) + 1 - p_2\right]
\end{aligned} \tag{S17}$$

with $M^* = 2M(1 - M)$. We thus get to the result presented in the main text:

$$\begin{cases}
\dfrac{\mathrm{d}p_1}{\mathrm{d}t} = 2M(1 - M)(1 - p_1 - p_2)[q_1(1 - s) - p_1(q_1 - s)] \\
\qquad\quad + p_1(1 - p_1)(q_1 - s) \\
\dfrac{\mathrm{d}p_2}{\mathrm{d}t} = 2M(1 - M)(1 - p_1 - p_2)[q_2 s - p_2(s + q_2 - 1)] \\
\qquad\quad + p_2(1 - p_2)(s + q_2 - 1)
\end{cases} \tag{S18}$$

S6.5 Coexistence solution

Let us assume there exists a fixed point of Eq. (S18), denoted (p_1^*, p_2^*), which is such that $0 < p_1^* < 1$ and $0 < p_2^* < 1$. This would correspond to a state of coexistence of the two varieties within both classes. We want here to find out under what conditions the existence of such a fixed point is not possible. We assume all parameters of the system are in the open unit interval. We found through symbolic computations that $q_1 \leq s$ prohibits its existence, but let us prove it by contradiction in a simple case, which is here the most physically relevant.

First, for $q_1 = s$:

$$\frac{\mathrm{d}p_1}{\mathrm{d}t} = 0 \Rightarrow p_1^* = 1 - p_2^*. \tag{S19}$$

It directly follows from the condition $\frac{\mathrm{d}p_2}{\mathrm{d}t} = 0$ that p_2^* must be either 0 or 1. In this case, there is therefore no possible coexistence.

Let us then assume $q_1 < s$. We have

$$\begin{aligned} \frac{\mathrm{d}p_1}{\mathrm{d}t} &= 0 \\ \Rightarrow 2M(1-M)(1-p_1^*-p_2^*)\left[q_1\frac{1-s}{s-q_1}+p_1^*\right] &= p_1^*(1-p_1^*). \end{aligned} \tag{S20}$$

As $s - q_1 > 0$ and $0 < p_1^* < 1$, both the right-hand side and the term in square bracket of the left-hand side are strictly positive. But we also have

$$\begin{aligned} \frac{\mathrm{d}p_2}{\mathrm{d}t} &= 0 \\ \Rightarrow 2M(1-M)(1-p_1^*-p_2^*) &= -\frac{p_2^*(1-p_2^*)(s+q_2-1)}{q_2 s - p_2^*(s+q_2-1)}. \end{aligned} \tag{S21}$$

Now, let us assume that $s + q_2 - 1 > 0$. It is the case that makes sense here, since variety 2, corresponding to standard language, has a higher prestige than 1, so $s > 0.5$, and at least a neutral bias of the high SE class, so $q_2 \geq 0.5$. Since $0 < p_2^* < 1$, this implies that the right-hand side above is negative, hence

$$2M(1-M)(1-p_1^*-p_2^*) < 0. \tag{S22}$$

This is in contradiction with the signs of the other terms in Eq. (S20). It is therefore strictly impossible to have coexistence solutions when $q_1 < s$ and $s + q_2 - 1 > 0$. In other words, for the non-standard form to survive, it is necessary that the SE class 1 has a positive bias towards the non-standard variety that is higher than the prestige of the standard form.

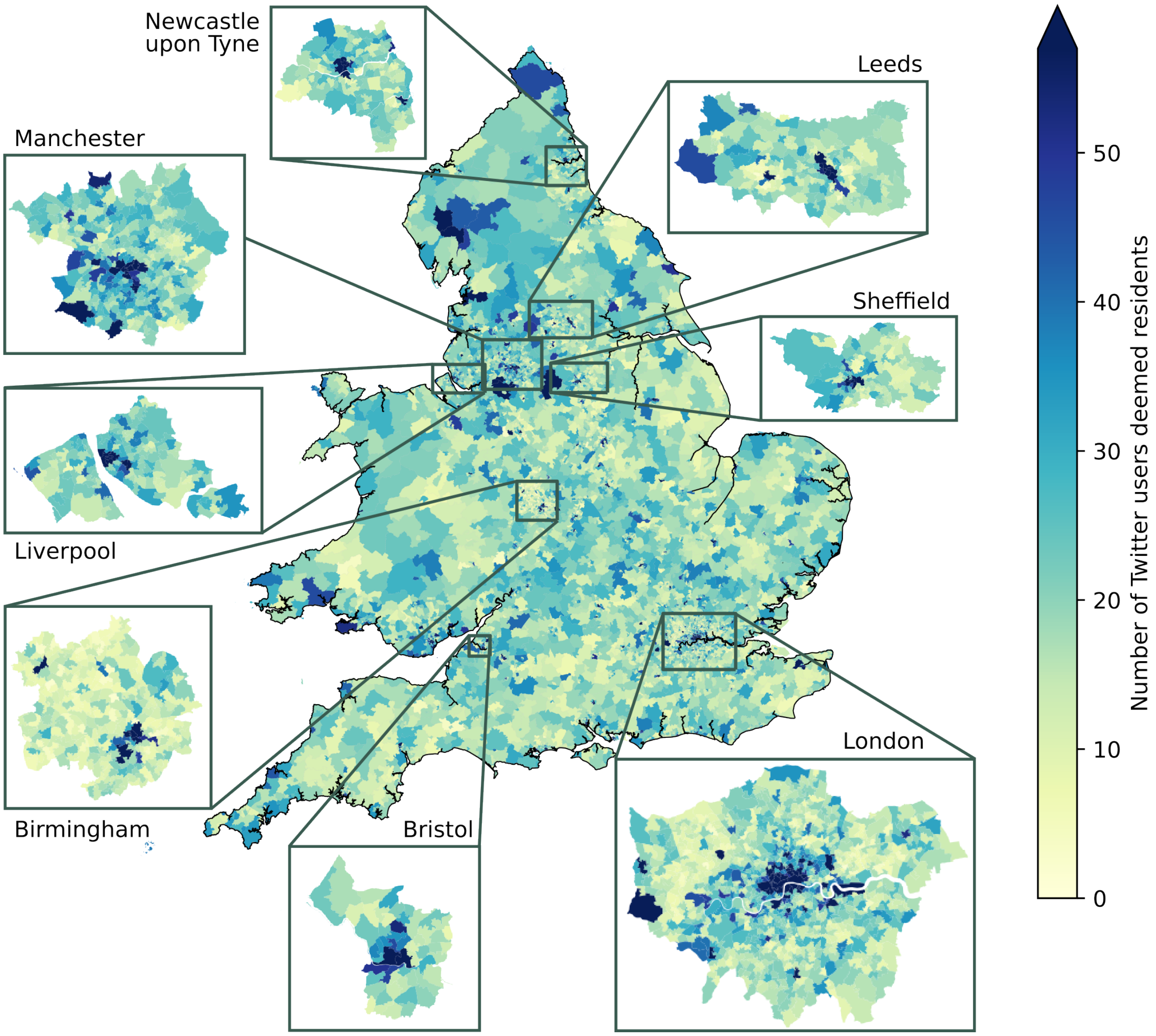

Figure S1: Twitter population map in England and Wales. The users counted in each MSOA were deemed residents of the areas. A zoom-in on each of the eight metropolitan areas of the study shows the MSOAs selected in their definition, which are also given in Table S3.

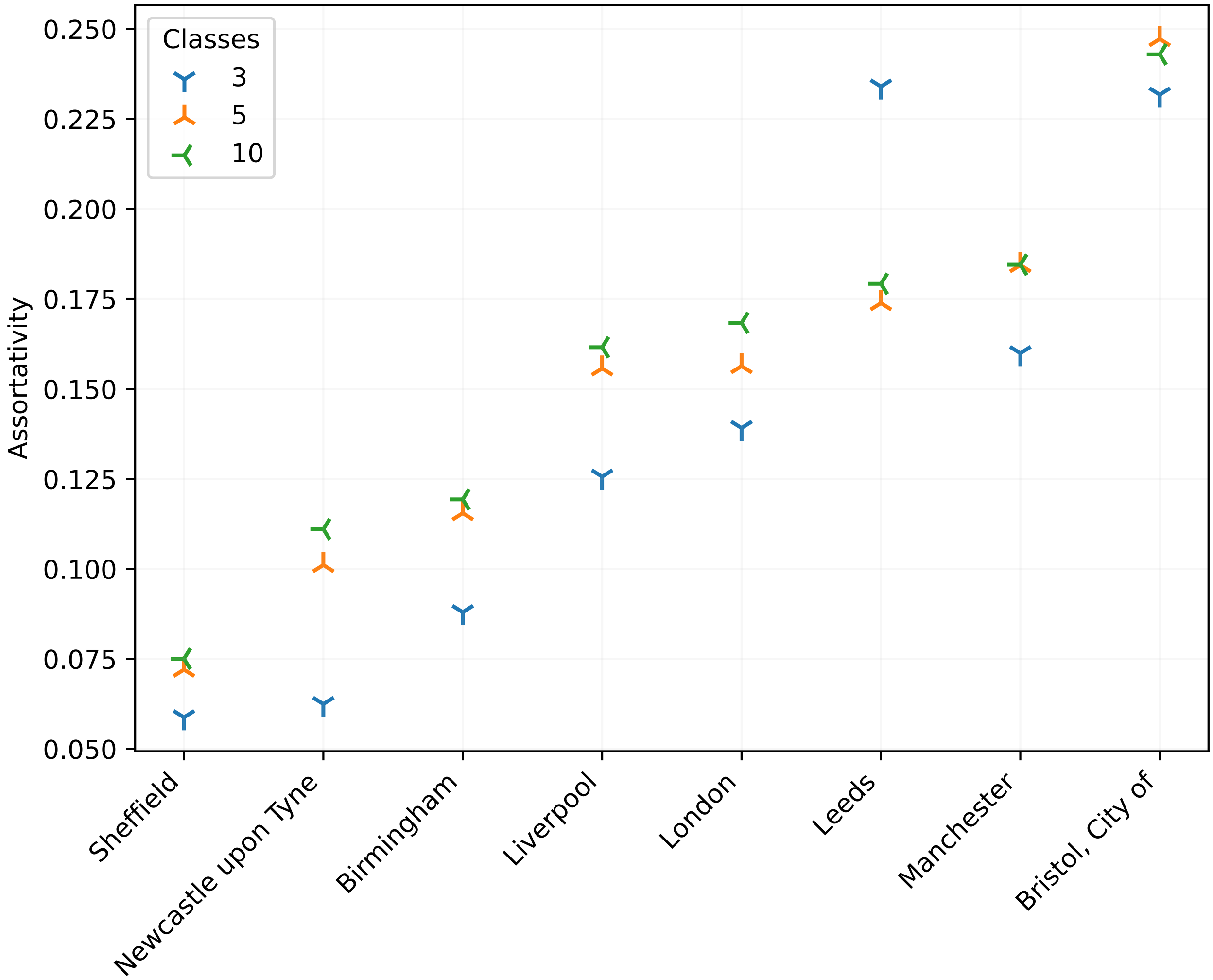

Figure S2: The influence of the number of classes on the computed assortativity.

Rule category	Count	Pearson r correlation with net income
Grammar	1 693 408	-0.25
Commonly Confused Words	283 731	-0.21
Possible Typo	1 977 466	-0.20
Miscellaneous	790 404	-0.12
Punctuation	3 525 135	-0.09
Style	708 659	-0.07
Compounding	88 391	-0.04
Collocations	46 814	-0.04
Nonstandard Phrases	5439	0.00
Redundant Phrases	103 643	0.02
Semantics	6425	0.03
Repetitions (Style)	1741	0.04
Upper/Lowercase	2 854 732	0.11
Typography	9 357 945	0.21

Table S1: Summary statistics about each category of rules defined by LanguageTool. The number of detected deviations from rules, as well as the Pearson r correlation with the net income in the 4879 MSOAs left after our filters.

Rule ID	Description	Count
HE_VERB_AGR	Agreement error: Non-third person/past tense verb with 'he/she/it' or a pronoun	122 203
THE_SUPERLATIVE	Zero or indefinite article ('a'/'an') before superlatives	69 461
PRP_VBG	He going (He is going)	58 775
CD_NN	Possible agreement error: numeral + singular countable noun	40 411
ITS_TO_IT_S	I have to do laundry while its (it's) still sunny	33 667
PHRASE_REPETITION	Repetition of two words ('at the at the')	33 414
PRP_PAST_PART	Agreement error: past participle without 'have'	32 913
A_NNS	Agreement: 'a' + plural word	32 902
BEEN_PART_AGREEMENT	Agreement: 'been' or 'was' + past tense	31 697
CAUSE_BECAUSE	confusion of cause vs. because	30 511

Table S2: Ten most frequently detected grammar rules. For each of them, we provide the rule ID assigned by LanguageTool, its description and their number of occurrences identified in the tweets of our home-located Twitter users.

Metropolitan area	LAD20CD	LAD20NM
Manchester	E08000001	Bolton
Manchester	E08000002	Bury
Manchester	E08000003	Manchester
Manchester	E08000004	Oldham
Manchester	E08000005	Rochdale
Manchester	E08000006	Salford
Manchester	E08000007	Stockport
Manchester	E08000008	Tameside
Manchester	E08000009	Trafford
Sheffield	E08000018	Rotherham
Sheffield	E08000019	Sheffield
Leeds	E08000032	Bradford
Leeds	E08000035	Leeds
Liverpool	E06000006	Halton
Liverpool	E08000011	Knowsley
Liverpool	E08000012	Liverpool
Liverpool	E08000015	Wirral
Birmingham	E08000025	Birmingham
Birmingham	E08000027	Dudley
Birmingham	E08000028	Sandwell
Birmingham	E08000030	Walsall
Birmingham	E08000031	Wolverhampton
Bristol, City of	E06000023	Bristol, City of
Newcastle upon Tyne	E08000023	South Tyneside
Newcastle upon Tyne	E08000024	Sunderland
Newcastle upon Tyne	E08000037	Gateshead
Newcastle upon Tyne	E08000021	Newcastle upon Tyne
Newcastle upon Tyne	E08000022	North Tyneside

Table S3: Definition of the metropolitan areas used in this study. For each metropolitan area, the code and the name of the local authority districts (LADs) contained within each area are given. London is defined as the London region (identified by the code E12000007).

Location	Tweets		Tokens		Deviations per token
	Sum	Per user	Sum	Per user	User average
Birmingham	770 000	275.9	10 613 724	3801.4	3.71×10^{-3}
Bristol, City of	373 245	243.9	5 218 801	3407.9	3.31×10^{-3}
Leeds	969 553	262.2	13 731 825	3710.9	3.52×10^{-3}
Liverpool	1 213 219	287.1	16 721 889	3954.6	3.81×10^{-3}
London	5 896 489	264.1	83 594 913	3741.0	3.24×10^{-3}
Manchester	2 496 379	288.5	35 370 104	4084.7	3.63×10^{-3}
Newcastle upon Tyne	1 051 460	287.9	14 691 320	4020.0	3.89×10^{-3}
Sheffield	619 512	287.9	8 924 594	4145.3	3.66×10^{-3}
England and Wales	37 376 633	264.8	520 730 958	3686.3	3.50×10^{-3}

Table S4: Summary statistics of our Twitter corpus. The number of tweets and tokens and their average number per user, as well as the frequency of non-standard features averaged over users are given for our eight metropolitan areas and all of England and Wales.

	Proportion of multilinguals:		
	Average	Correlation with grammar deviations	p-value
Birmingham	3.8%	−0.12	0.26
Bristol, City of	4.3%	−0.26	0.10
Leeds	2.9%	0.08	0.44
Liverpool	3.9%	0.02	0.83
London	7.6%	0.00	0.94
Manchester	3.5%	0.03	0.67
Newcastle upon Tyne	4.5%	−0.14	0.14
Sheffield	4.2%	−0.06	0.62

Table S5: Proportions of multilinguals among our identified residents and their correlation with grammar deviations.
